# The role of surgical audit in improving patient management; nasal haemorrhage: an audit study

**DOI:** 10.1186/1471-2482-7-19

**Published:** 2007-09-13

**Authors:** Tahwinder Upile, Waseem Jerjes, Fabian Sipaul, Mohammed El Maaytah, Seyed Ahmad Reza Nouraei, Sandeep Singh, Colin Hopper, Anthony Wright

**Affiliations:** 1 Royal National Institute of Throat Nose and Ear Hospital, London, UK; 2 Oral & Maxillofacial/Head & Neck Unit, University College London Hospitals, London, UK; 3 Unit of Oral & Maxillofacial Surgery, Division of Maxillofacial, Diagnostic, Medical and Surgical Sciences, UCL Eastman Dental Institute, London, UK; 4 Department of Surgery, Royal Free & University College Medical School, London, UK

## Abstract

**Background:**

Nasal bleeding remains one of the most common Head & Neck Surgical (Ear Nose and Throat [ENT]/Oral & Maxillofacial Surgery [OMFS]) emergencies resulting in hospital admission. In the majority of cases, no other intervention is required other than nasal packing, and it was felt many cases could ideally be managed at home, without further medical interference. A limited but national telephone survey of accident and emergency departments revealed that early discharge practice was identified in some rural areas and urban departments (where adverse socio-demographic factors resulted in poor patient compliance to admission or follow up), with little adverse patient sequelae. A simple nasal packing protocol was also identified.

The aim of this audit was to determine if routine nasal haemorrhage (epistaxis) can be managed at home with simple nasal packing; a retrospective and prospective audit.

Ethical committee approval was obtained. Similar practice was identified in other UK accident and emergency centres. Literature was reviewed and best practice identified. Regional consultation and feedback with regard to prospective changes and local applicability of areas of improved practice mutually agreed upon with involved providers of care.

**Methods:**

Retrospective: The Epistaxis admissions for the previous four years during the same seven months (September to March).

Prospective: 60consecutive patients referred with a diagnosis of Nasal bleeding over a seven month time course (September to March). All patients were over 16, not pregnant and gave fully informed counselled consent.

New Guidelines for the management of nosebleeds, nasal packing protocols (with Netcel^®^) and discharge policy were developed at the Hospital. Training of accident and emergency and emergency ENT staff was provided together with access to adequate examination and treatment resources. Detailed patient information leaflets were piloted and developed for use.

**Results:**

Previously all patients requiring nasal packing were admitted. The type of nasal packing included Gauge impregnated Bismuth Iodoform Paraffin Paste, Nasal Tampon, and Vaseline gauge. Over the previous four year period (September to March) a mean of 28 patients were admitted per month, with a mean duration of in patient stay of 2.67 days.

In the prospective audit the total number of admissions was significantly reduced, by over 70%, (χ^2 ^= 25.05, df = 6, P < 0.0001), despite no significant change in the number of monthly epistaxis referrals (χ^2 ^= 4.99, df = 6, P < 0.0001). There was also a significant increase in the mean age of admitted patients with epistaxis (χ^2 ^= 22.71, df = 5, P < 0.0001), the admitted patients had a mean length of stay of 2.53 days. This policy results is an estimated saved 201.39 bed days per annum resulting in an estimated annual speciality saving of over £50,000, allowing resource re-allocation to other areas of need. Furthermore, bed usage could be optimised for other emergency or elective work.

**Conclusion:**

Exclusion criteria have now been expanded to exclude traumatic nasal haemorrhage. New adjunctive therapies now include direct endoscopic bipolar diathermy of bleeding points, and the judicious use of topical pro-coagulant agents applied via the nasal tampon. Expansion of the audit protocols for use in general practice.

This original audit informed clinical practice and had potential benefits for patients, clinicians, and provision of service. Systematic replication of this project, possibly on a regional and general practice basis, could result in further financial savings, which would allow development of improved patient services and delivery of care.

## Background

One of the biggest threats to waiting list times is the habitual occupancy of beds by emergency admissions. We wished to determine if surgical audit would have utility in improving local surgical care in the management of epistaxis and reduce unnecessary admission rate. Nasal bleeding remains one of the most common ENT and OMFS emergencies resulting in hospital admission [1], quoted approximately at 30 per 100000 adults [2]. Its cause tends only to be identified in 15% of patients, the remainder classified as idiopathic. In the majority of cases no other intervention is required other than nasal packing, and it was felt many cases could ideally be managed at home, without further medical interference. In fact, before the introduction of the National Health Service (NHS), epistaxis was largely managed at home with only the most severe cases referred for admission [3,4].

A telephone survey of several UK accident and emergency departments revealed that early discharge practice in some rural areas (Isle of Wight) and urban departments (Leeds), where presumably adverse socio-demographic factors may have resulted in poor patient compliance to admission or follow up, with little adverse patient sequelae. Literature on epistaxis management was reviewed [5,6] and best practice identified [7-10]. It was determined that simple nasal packing was the only required medical intervention in the vast majority of cases of nosebleeds not responding to first aid measures. Furthermore, a simple but efficacious non-specialist nasal packing protocol was also identified [7-9]. Regional consultation and feedback with regard to prospective changes and local applicability of areas of improved practice mutually agreed upon with involved providers of care (General Practitioners [GPs], Accident & Emergency [A&E] Staff, ENT and OMF Surgeons) was undertaken. Research and development, local ethics committee and hospital audit committee approval was subsequently granted.

## Methods

Detailed reviews of past Nasal Haemorrhage admissions for the previous four years was undertaken in the form a review of notes, admission details and bed usage.

New Guidelines for Epistaxis management and nasal packing protocols using nasal tampons (e.g. Netcel^® ^Polyvinyl alcohol sponge) and discharge policies were developed in the ENT, OMFS and A&E Departments. Training of A&E and ENT/OMFS staff was provided together with access to adequate examination and treatment resources. Detailed patient information leaflets were piloted and developed for use. The audit trial was conducted for 7 months (September to March) and compared to data collected during the same time interval over (September to March)the last 4 years to adjust for seasonal variations in presentation and admission. The study group consisted of all patients over 16, who were not pregnant and who gave fully informed counselled consent. 60consecutive patients were referred to the ENT Department with a diagnosis of Epistaxis over a seven-month time course (September to March) who matched the inclusion criteria. They either presented to the A&E Department or referred by GPs to the ENT Department. Patients seen by either the A&E or the ENT staff of other hospitals were excluded.

A proforma was created for the A&E Department regarding the management of epistaxis, which was enacted by both the medical personnel and the emergency nurse practitioners. All epistaxis patients were resuscitated as required according to the Advanced Trauma Life Support (ATLS) protocol. First aid measures were instituted i.e. pinching the whole of the cartilaginous tip of the nose for 30 minutes followed by another 30 minutes of pressure and pack of ice on bridge of nose if bleeding continued. If the epistaxis persisted, blood and clot was removed to try and visualize the bleeding vessel. Cautery was then attempted. In the event the bleeding continued, local anaesthetic in the form of Lidocaine Hydrochloride 5% and Phenylephrine Hydrochloride 0.5% was sprayed into the nose, left for 5–10 minutes and Netcell^® ^nasal pack was inserted into the nose and to the other side as well if necessary.

The pack was lubricated with KY Jelly prior to insertion and activated by 10–15 mls of cold tap water. They were then observed for at least 30 minutes. Failure of cessation of bleeding by this time is an absolute criterion for referral to the ENT team for possible admission. The other absolute criteria included shock, nasal packing within the last 7 days, anticoagulant medication, Haemoglobin of less than 10 g/dl and uncontrolled hypertension. There were also relative criteria for referral e.g. home more than 20 minutes away from the hospital, attendance to the hospital with epistaxis within the last 24 hours, living alone, no car or telephone and if unhappy to be discharged. A contact name and number was supplied on discharge. Monthly audit of proforma and patient outcomes was also undertaken during the audit to identify problem areas.

## Re-evaluation

One patient had to be excluded after being inadequately packed in the accident department and was subsequently managed by bipolar diathermy in the ward and sent home the same day.

Forty-four patients were allowed home with nasal packing. Fifteen patients had to be admitted on presentation; of which 9 were admitted as they oozed persistently after packing with Netcel, 1 was admitted after sustaining trauma to the nose and 3 were not happy to be discharged. One admission experienced vaso-vagal syncope in casualty and was admitted by a locum Senior House Officer (SHO) who was not aware of the study.

**Figure 3:**
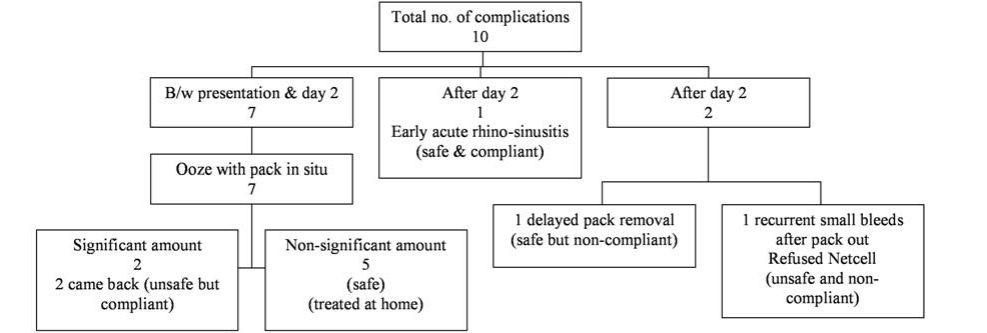
Review of the complications encountered in patients who underwent nasal packing and who were allowed home. Nearly all the patients could be managed on an outpatient or 'office' basis. Unfortunately, one patient who suffered a nosebleed after an alcohol related trauma had to be admitted because a failure of compliance. We subsequently revised the treatment protocol after a multi-disciplinary meeting to exclude traumatic epistaxis where management may have to be surgical in the case of vessel retraction following fracture transaction.

There were a total of 10 complications (Figure 3). Seven patients had bloody nasal oozing between day one and day two; of these 2 returned to the ward one of whom had nasal cautery with AgNO3 and was allowed home, while the other was admitted. Five patients reported bloody nasal oozing at home, which stopped with first aid measures. At day two, one patient complained of symptoms suggestive of early acute rhino-sinusitis which settled spontaneously. There were 2 patients with complication after day two, of these one patient did not get an advice sheet and only came back to have the pack removed at day 7, whilst the other patient had recurring minor epistaxis after pack removal. This patient was a healthy non compliant 18 years old male who had traumatic epistaxis 3 days prior to presentation. He was appropriately packed and given the advice sheet and allowed home. His Haemoglobin at presentation was 13.5 g/dl, the next day he had pack removal with no further bleeding but at day 3 he returned with recurrent minor epistaxis. Unfortunately he refused further packing and admission. He was seen in clinic at day 6 and was found to be anaemic with Haemoglobin of 7.3 g/dl. He was counselled and subsequently received a blood transfusion and did not experience any further epistaxis. As a result of these complications the exclusion criteria and treatment sheets for the audit were modified.

## Results

Previously all patients requiring nasal packing were admitted. The type of nasal packing included Gauze impregnated Bismuth Iodoform Paraffin Paste (BIPP), Nasal Tampon, and Vaseline gauze. During the previous four years and over the same 7 month period (September to March) a mean of 28 patients were admitted per month, with an average in patient stay of 2.67 days.

**Figure 1:**
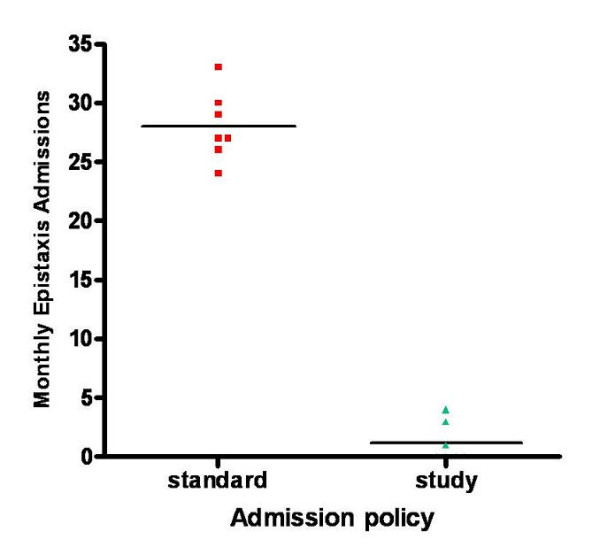
Number of monthly admissions with nasal haemorrhage in the standard (mean monthly admissions over the previous 4 years; small square points) and audit study (small triangular points) over the 7-month (September-March) time interval to allow for seasonal variations. This shows a significant reduction in the numbers of admissions.

**Figure 2:**
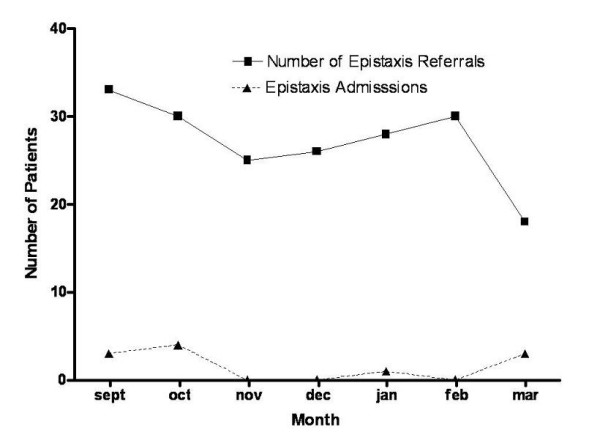
Total of monthly referrals and admissions with epistaxis during audit study over the 7-month (September-March) intervention time interval. It should be noted that previously nearly all referrals of epistaxis patients requiring packing had to bee admitted. It can be seen that this was not the case for the study period.

The method of nasal packing was well tolerated by patients with over 76% of patients having a visual analogue pain score of 6/10 or less at insertion. There were no patient complaints with regard to the discharge policy. Analysis by a Wilcoxon signed rank test suggested a significant difference (p < .01) between the number of admissions between the audit protocol group and standard admission policy group (Figure 1). In this prospective audit the total number of admissions was significantly reduced by 73% (χ^2 ^= 25.05, df = 6, P < 0.0001), despite no significant change in the number of monthly referrals (χ^2 ^= 4.99, df = 6, P < 0.0001) (Figure 2). There was also a significant increase in the mean age of admitted patients with epistaxis (χ^2 ^= 22.71, df = 5, P < 0.0001), the admitted patients had a mean length of stay of 2.53 days (Table 1). This policy has resulted in an estimated 201.39 saved bed days per annum for the hospital resulting in an estimated annual saving of over £52,632, allowing resource re-investment and re-allocation to other areas of need.

**Table 1:** The patients' demographics

Demographics	Discharged	Admitted
Number (%)	73	27
Age (mean years)	67	72
**Male :Female (n)**	**29:15**	**6:10**
Using Anticoagulation	34%	56%
Pulse (mean)	86 bpm	91 bpm
Systolic BP	159	156
Diastolic BP	91	93

## Further audit spiral

We now formally require patient's signature on the treatment sheet to indicate that they have read and understood the advice sheet. Exclusion criteria have now been expanded to exclude traumatic epistaxis that does not cease bleeding after simple first aid measures (Figure 3). New adjunctive therapies now include direct endoscopic bipolar diathermy of bleeding points [9,10], and the judicious use of topical pro-coagulant agents applied via the nasal tampon. Expansion of the audit protocols for use in general practice is also underway.

## Discussion

The vast majority of cases of epistaxis are dealt by A&E personnel or the ENT/OMFS SHOs with little experience. Nasal packing with a nasal tampon or similar material is the technique of choice for these personnel [5]. Netcell^® ^nasal pack was chosen over BIPP because was reported to be easier to use and insert than BIPP in inexperienced hands. The technique involved for effective packing requires minimal skill and ENT knowledge. It can be inserted blindly without the need for special equipment or adequate lighting. The pack is made of Polyvinyl Alcohol, which reportedly promotes platelet aggregation, and does not support bacterial or fungal growth. It is fibre-free and this minimises trauma to the nasal mucosa. It is less complicated and more acceptable to both patients and A&E personnel and avoids iodine allergy or intolerance. Importantly there is no significant difference in term of effectiveness in arresting epistaxis.

Tranexamic acid was used as an adjunct in some patients with persistent ooze. It was injected directly into the pack. It is primarily used in intravenously in patients with intractable menorrhagia. The evidence in epistaxis is still only anecdotal at present. In our study it seemed to have helped stem the bleeding in all the three cases it was used.

We did not routinely prescribe antibiotic. In the face of increasing antibiotic resistance and lack of evidence to show that routine antibiotic in epistaxis patients with nasal pack in-situ for less than 48 hours makes any difference, we felt this was inappropriate.

Anticoagulation did not appear to be a barrier to discharge management if the level of anticoagulation was within the therapeutic goal for the patients and his pre-existing pathology. Aspirin did not seem to be a major determinant in deciding whether patients were going to be admitted or not. Age did seem to be an important predictive factor for admission. We compared the age difference of the persistent bloody nasal dischargers in the admitted group with those sent home. Those whom were deemed to be safe to be sent home had an average age of almost a decade younger (66.3). Blood pressure on presentation however did not seem to be much different in the two groups mentioned.

There was only 1 significant complication in this study, requiring readmission and formal surgical cautery. This was mainly due to the patient not being fully compliant (Figure 3). The key to the success of the study appeared to be the advice sheet, this contained advice on how to minimise recurrence of epistaxis and on what to do if the epistaxis persist despite first aid steps.

Forty-four patients (73%) were safely managed at home with Netcell^® ^nasal packs and avoided admissions. In our hospital the average length of admission for epistaxis was 2.67 days and thus projecting annually, potentials of 201.39 bed days were saved.

## Conclusion

After careful assessment and provided that the patient's nose is properly packed with a nasal sponge tampon and advice sheet is given and understood, we believe it is safe to manage selected patients with routine epistaxis at home.

We feel that surgical audit does have utility in improving local surgical care in the treatment of epistaxis. This original audit informed our clinical practice and had potential benefits for patients, clinicians, and provision of service. We hope that a systematic replication of this project, possibly on a regional and general practice basis, could result in further financial savings, which would allow development of improved patient services and delivery of care.

## Competing interests

The author(s) declare that they have no competing interests.

## Authors' contributions

TU designed the study, carried out the literature research, clinical study and manuscript preparation. WJ designed the study, carried out the literature research, manuscript preparation, and manuscript review. SF designed the study, carried out the literature research, manuscript preparation, and manuscript review. ME carried out the manuscript editing and manuscript review. RN carried out the manuscript editing and manuscript review. SS carried out the manuscript editing and manuscript review. CH carried out the manuscript editing and manuscript review. AW designed the study, carried out the literature research, clinical study and manuscript preparation.

All authors read and approved the final manuscript.

## Pre-publication history

The pre-publication history for this paper can be accessed here:



## References

[B1] Viehweg TL, Roberson JB, Hudson JW (2006). Epistaxis: diagnosis and treatment. J Oral Maxillofac Surg.

[B2] Juselius H (1974). Epistaxis. J Laryngol Otol.

[B3] Hippocrates (5th Century BC), Translated by Adams F (1877). St Petersburg Med Wchnschr.

[B4] Chaiyasate S, Roongrotwattanasiri K, Fooanan S, Sumitsawan Y (2005). Epistaxis in Chiang Mai University Hospital. J Med Assoc Thai.

[B5] Klossek JM, Dufour X, de Montreuil CB, Fontanel JP, Peynegre R, Reyt E, Rugina M, Samardzic M, Serrano E, Stoll D, Chevillard C (2006). Epistaxis and its management: an observational pilot study carried out in 23 hospital centres in France. Rhinology.

[B6] Corbridge RJ, Djazaeri B, Hellier WP, Hadley J (1995). A prospective randomized controlled trial comparing the use of merocel nasal tampons and BIPP in the control of acute epistaxis. Clin Otolaryngol.

[B7] Annys E, Jorissen M (2000). Short term effects of antibiotics (Zinnat) after endoscopic sinus surgery. Acta Otorhinolaryngol Belg.

[B8] McGarry GW, Moulton C (1993). The first aid management of epistaxis by accident and emergency department staff. Arch Emerg Med.

[B9] O'Donnell M, Robertson G, McGarry GW (1999). A new bipolar diathermy probe for the outpatient management of adult acute epistaxis. Clin Otolaryngol.

[B10] Van Wyk FC, Massey S, Worley G, Brady S (2006). Do all epistaxis patients with a nasal pack need admission? A retrospective study of 116 patients managed in accident and emergency according to a peer reviewed protocol. J Laryngol Otol.

